# Rectal Irrigation in Enterocolitis of Children

**Published:** 1906-09

**Authors:** Clarence G. Clarke

**Affiliations:** New York City


### RECTAL IRRIGATION IN ENTEROCOLITIS OF CHILDREN.

BY CLARENCE G. CLARKE, M.D.,

NEW YORK CITY.

When we consider that nearly one-half of all the children born die before the age of one year, and that by far the greater portion of these die from some form of intestinal indigestion, we realize what an immense field there is for the improvement of our technique in the treatment of these affections. Our infantile mortality could undoubtedly be greatly decreased could all cases be seen and treated actively from the outset. Although a great many are cases of a violent character from the very outset, still there is a certain proportion of a more or less chronic nature, and it is this class of cases which greatly tax the ingenuity and patience of the physician. Mo medication by the mouth seems efficient to stop the progressive wasting. As soon as food is taken it is passed out without the child absorbing any of it. It is of one of these cases that I wish to present to the medical profession a report, hoping that the methods adopted will be found successful in other cases of a similar nature.

Baby D., age four months. History: Child was nursed by mother for one and a half months, but as her milk was of poor quality and child did not thrive, she was advised by her attending physician to stop the breast and substitute bottle. This she did, feeding the child on a mixture of milk, cream, milk, sugar and barley water in a 3-6-1 proportion. The baby thrived on this for about two months, but early in June it developed a diarrhea. The mother gave it home remedies but still continued the milk, feeding it even more frequently than before, as the child was fretful and apparently hungry. The stoll averaged seven to eight a day, and occasionally the child would vomit. This continued for two weeks, when the mother became worried at the progressive emaciation and decided to call a physician. I first saw the case on July 16th.

*Examination.*—Baby extremely emaciated; lies on cot without apparent strength to move; tongue fissured; cheeks sunken; abdomen tympanitic (slight); temperature 102.2; weight seven pounds; stools ten to twelve daily, full of mucus and curds of undigested milk. I gave the mother a very unfavorable prognosis, but told her to secure a nurse for the baby, and we would do all that was possible.

*Treatment.*—Milk was stopped at once. Child fed on barley water and albumen water. I then ordered the nurse to wash out the colon twice daily through a catheter with two quarts of a solution containing glyco-thymoline one part, and water ten parts. By the mouth I have 1/20 gr. calomel tablets every hour for ten hours, and twenty drops of brandy every two hours.

July 17th: Child in about the same condition, except that it had only nine stools in twenty-four hours, and they were of a trifle better color with less mucus and no curds. Continued irrigations, but stopped calomel. Continued with brandy.

July 18th: Seven stools, quite watery but of a much better color. Treatment continued until July 24th, at which time the child was much improved, having only three in a day and passing very little mucus. On this date I started the milk again, using a very dilute formula with three ounces of milk sugar and fifteen ounces of boiled water. Continued irrigation with glyco-thymoline, one to eight ounces a day, but stopped all other medicine. The baby started to thrive at once, and in two weeks more we again weighed the child and noted an increase of three pounds. I gradually increased the strength of his food until at the present time he is taking eight ounces of milk to eleven ounces of water and one ounce of lime water, which is almost the average for a child of his age (five and one-half months).

This is only one case of a number that I have treated with nearly the same routine this summer, and all with satisfactory results. I lay the success I have had to two factors: First, the immediate withdrawal of all milk, and, second, the continuous and copious irrigation. For this irrigation I have tried numerous solutions, but nothing to equal glyco-thymoline in a one to ten proportion. It appears to cleanse the inflamed colon better than anything else, and in nearly all cases of this nature I have had quick improvement in the character of the stools after its use.

In conclusion, I would state, that although in this case I did not give much treatment by mouth, because the symptoms seemed to point more to a lower-bowel affection, yet in many cases where gastric symptoms have been more predominant I have combined with the irrigation treatment glyco-thymoline in 15-30 m. doses combined with liquor bismuth as follows:

| | | |
|---|---|---|
| ℞ | Glyco-Thymoline | ʒ ss. |
| | Liq. Bismuth | ʒ i. |
| | Aqua | ʒ ii. |

M. ʒ i. q. 2-3 h.

This, in connection with rectal irrigation with glyco-thymoline, in proportion indicated, will suffice in nearly all the cases of gastro-enteritis, enterocolitis and enteritis so common in artificially fed infants.

### Affections of the Thyreoid Gland.

Beilby thinks operation is rarely undertaken for the deformity alone. The severity of the symptoms is the guide for treatment. Inasmuch as secondary changes in these tumors are very frequent, they should be considered on the same basis as similar lesions in other organs. In simple hypertrophy, if the gland continues to enlarge, a portion of it should be removed. If a new growth recurs, especially if it is suggestive of cancer, operation may be imperative for the relief of pressure symptoms. Kocher's vast experience with thyreoidectomy shows that under local anesthesia as low a mortality as 0.4 per cent. is possible. Partial thyreoidectomy is becoming the method of choice in exophthalmic goitre. Good results in this condition have been reported from the use of the milk of thyreoidectomized goats, also from the serum of thyreoidectomized sheep. An early operation is desirable, before damage has occurred to the nervous system. The importance of local anesthesia for this operation is also noteworthy.—*Annals of Surgery, June, 1906.*

